# Resource use and costs of transitioning from pediatric to adult care for patients with chronic kidney disease

**DOI:** 10.1007/s00467-023-06075-w

**Published:** 2023-07-19

**Authors:** Daniela Choukair, Susanne Rieger, Dirk Bethe, Dorothea Treiber, Georg F. Hoffmann, Corinna Grasemann, Peter Burgard, Jörg Beimler, Janna Mittnacht, Burkhard Tönshoff

**Affiliations:** 1 Department of Pediatrics I, University Children’s Hospital Heidelberg, Heidelberg, Germany; 2 Center for Rare Diseases, University Hospital Heidelberg, Im Neuenheimer Feld 130.3, 69120 Heidelberg, Germany; 3 Department of Pediatrics, St-Josef Hospital Bochum and Center for Rare Diseases, Ruhr-University Bochum, Bochum, Germany; 4 Department of Nephrology, University Hospital Heidelberg, Heidelberg, Germany

**Keywords:** Chronic kidney disease, Costs, Empowerment, Health literacy, Kidney transplantation, Transition from pediatric to adult care

## Abstract

**Background:**

The structured transition of adolescents and young adults with chronic kidney disease (CKD) from pediatric to adult care is important, but data on the time and resources required for the necessary components of the transition process and the associated costs are lacking.

**Methods:**

In a prospective single-center cohort study of 52 patients with pre-transplant CKD (CKD stage 1, *n* = 10; stage 2, *n* = 6; stage 3, *n* = 5; stage 4 and 5, 1 patient each) or kidney transplant recipients (KTR), resource use and costs were evaluated for the key elements of a structured transition pathway, including (i) assessment of patients’ disease-related knowledge and needs, (ii) required education and counseling sessions, and (iii) compiling an epicrisis and a transfer appointment of the patient with the current pediatric and the future adult nephrologist.

**Results:**

Forty-four of 52 enrolled patients (84.6%) completed the transition pathway and were transferred to adult care. The mean time from the decision to start the transition process until the final transfer consultation was 514 ± 204 days. The process was significantly longer for KTR (624 ± 150 [range, 307–819] days) than for patients with pre-transplant CKD (365 ± 172 [range, 1–693] days; *P* < 0.0001). The cumulative costs of all counseling and education sessions performed including the transfer appointment were 763 ± 473 Euro; it was significantly higher in KTR (966 ± 457 Euro) than in patients with pre-transplant CKD (470 ± 320 Euro; *P* < 0.0001).

**Conclusions:**

A structured transition pathway for patients with CKD is resource and time–consuming due to the complexity of the disease and should be sufficiently funded.

**Graphical Abstract:**

**Figure:**
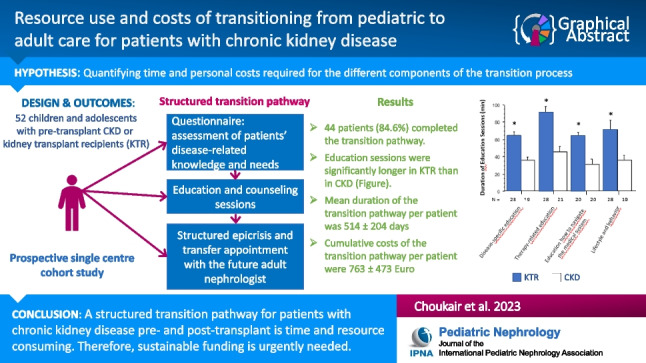
A higher-resolution version of the Graphical abstract is available as Supplementary information

**Supplementary Information:**

The online version contains supplementary material available at 10.1007/s00467-023-06075-w.

## Introduction

Adolescents with chronic diseases must move from child-centered to adult-centered health care systems during a special period of change [1, 2]. This process is referred to as transition, while the transfer is the discrete event marking the movement of the patient from pediatric care to the adult health care system [3, 4]. However, even among adolescents with chronic conditions who are receiving regular medical care, the transfer to a specialized adult care setting often does not occur [4]. For example, in at least 40% of adolescents with inflammatory bowel disease and with type 1 diabetes, the end of care in pediatric structures marks a break in medical care with negative consequences for adherence, deterioration of symptom control, development of secondary diseases, or even the development of irreversible organ damage [5, 6]. For example, non-adherence during and after transfer from pediatric to adult transplant units resulted in kidney transplant failure in 35% of patients [7]. Neurodevelopment is still ongoing when most patients are transferred, and many have not yet mastered all of the tasks required for independent self-management [8]. To address these challenges, several transition programs have been designed and implemented worldwide, mostly at the local level and often focused on a specific disease or group of diseases [9]. For patients with chronic kidney disease (CKD), the International Society of Nephrology (ISN) and the International Pediatric Nephrology Association (IPNA) have developed a consensus statement focusing on patient education, empowerment, and autonomy [10]. However, these efforts are often underfunded in national health systems and therefore not widely or sustainably implemented [11, 12].

To improve the care of patients with rare diseases (RD), the German Federal Joint Committee (G-BA) funded the TRANSLATE NAMSE innovation project from April 2017 to September 2020 [13, 14]. Ten German centers for Rare Diseases, two health insurance companies (AOK Nordost; Barmer GEK), and the Alliance for Chronic Rare Diseases (ACHSE e.V.) established a consortium to design, test, and evaluate a model for structured care of patients with RD [15]. The TRANSLATE-NAMSE project developed a generic pathway and supporting instruments which can be applied to adolescents with different RD [16]. This pathway is based on previous experience with adolescents with CKD in our center [17] and the “Berlin Transition Program” (BTP) [18]. It designs the transition process for a period of 2 years by providing a framework of transition consultations, a transition booklet, and a structured summary of the hitherto course of the disease (epicrisis), orchestrated by a case manager, who ensures that the enrolled patients stay within the program and do not get lost, thus ensuring treatment continuity. Here, we describe the transition pathway and report the results for participating patients with pre-transplant CKD and kidney transplant recipients (KTR) at the University Children’s Hospital Heidelberg, Germany. The specific objectives were to quantify the time required for the different components of the transition process and the corresponding personnel costs for the different members of the multidisciplinary team.

## Methods

A prospective cohort of 52 patients with pre-transplant CKD or KTR was recruited between December 1, 2017, and February 28, 2020, at the Pediatric Nephrology Outpatient Clinic of the University Children’s Hospital Heidelberg. This cohort was part of the nationwide health care project entitled TRANSLATE NAMSE funded by the Innovation Fund of the German Federal Joint Committee (G-BA), grant number 01NVF16024 TRANSLATE NAMSE [13, 14]. The study was approved by the Ethics Committee of the Charité, Berlin (#EA2/140/17) and the Ethics Committee of the University Hospital Heidelberg (S-499/2017). Written informed consent was obtained from all parents/guardians, with assent from patients when appropriate for their age. Inclusion criteria were as follows: (i) pre-transplant CKD or KTR, (ii) age ≥ 16 years, (iii) willingness to participate in a structured transition program with expected transfer to adult care within 2 years. Exclusion criteria were as follows: (i) severe intellectual disability and (ii) lack of informed consent.

### Transition pathway

The transition pathway was developed as part of the national health care project TRANSLATE-NAMSE and had the following aims: (i) to establish standardized and transparent care processes, (ii) to establish the standardized case and care management, (iii) to establish interdisciplinary networking and an information exchange among experts, (iv) to increase health literacy through education and counseling [19], (v) to sustainably improve the care of transition patients with rare diseases, (vi) to increase knowledge and information transfer through information on a care pathway and, (vii) to avoid graft loss in KTR after the transfer (Supplemental Fig. 1) [16]. At the beginning of the transition process, patients received a standardized questionnaire from their pediatric nephrologist. The standardized questionnaire includes 30 items on (A) disease-specific knowledge, (B) management of medications and other treatment modalities, (C) social support and information, (D) future and career planning, (E) autonomous navigation through the medical system, and (F) wishes to the care team (Supplemental Table 1). The level of information as well as education and counseling needs were quantified using an Excel-based calculation sheet. The item responses were coded as follows: 0, agree; 0.5, partially agree; 1, disagree. For example, the domain “disease-specific knowledge” contained 6 items. A single entry of 0.5 corresponded to 8.3%, and a single entry of 1 corresponded to 16.66% of a maximum of 6 items. All entries were cumulated and expressed as the percentage of 6, representing the disease-specific counseling need. Based on this assessment, the multidisciplinary care team provided health literacy education to patients in areas of identified knowledge and competence gaps. The educational programs performed are described in detail in a previous manuscript by our group [17]. Additional appointments were provided for psychological, social-legal, and genetic counseling as indicated by the patient or deemed necessary by the pediatric nephrologist. Seventeen of 52 patients (32.7%) of this study population had a migration background. Where there was an obvious language barrier, the training and counseling sessions were conducted with the help of an interpreter. Depending on need, 1–5 h of transition counseling was available per patient. The attending pediatric nephrologist prepared a structured epicrisis including recommendations for further management.

Upon completion of the counseling and education sessions, the case manager scheduled an appointment for the actual transfer of the patient to adult care, preferably with both the pediatric and adult nephrologists. This meeting included the following: (i) introducing new care providers, (ii) handing over all relevant patient information including molecular genetic results, (iii) explaining the adult care process, (iv) handing over the written epicrisis to the adult nephrologist, (v) providing the patient with the contact details of the adult care team in case of emergencies. If it was not possible to organize a transition consultation together with the adult care team, the future adult nephrologist received the relevant documents, and the important patient information was discussed with him by phone. The transition process was completed with a final consultation in the pediatric clinic. A follow-up appointment was scheduled to prevent patients from losing contact. The initiation, duration, and completeness of this patient pathway, the quantified level of disease-specific knowledge, and the hours of education and counseling provided were documented in a checklist.

### Calculation of personnel costs

The calculation of personnel costs, based on the German staff remuneration rates for the years 2017–2019, is shown in Supplemental Table 2. For further calculations, we used the following respective personnel costs per minute: pediatric or adult nephrologist, 0.72 Euro/min; psychologist, 0.59 Euro/min; social worker, 0.52 Euro/min; dietician, 0.48 Euro/min; nurse, 0.46 Euro/min. The costs of the transition consultation were calculated as follows: the duration of the consultation in minutes multiplied by the costs in minutes of the participating members of the multidisciplinary team. The costs of all consultations per patient were calculated as the sum of the costs of all transition consultations per patient. The average costs of a consultation per patient were calculated as the cost of all transition consultations per patient divided by the number of consultations.

### Statistical analysis

Patient data were collected in portable document format (PDF), which were read as comma-separated values (CSV) files and imported into SPSS 26 (SPSS Inv., Chicago, IL, USA), checked for plausibility and completeness, and analyzed descriptively. Data were tested for normal distribution using the Kolmogorov–Smirnov test. Data are presented as mean ± standard deviation (SD) or as median and range. The unpaired Student *t*-test was used to compare normally distributed groups, and the Wilcoxon–Mann–Whitney test was used to compare non-normally distributed groups. A *P* value < 0.05 was considered statistically significant.

## Results

### Patient characteristics

**Fig. 1:**
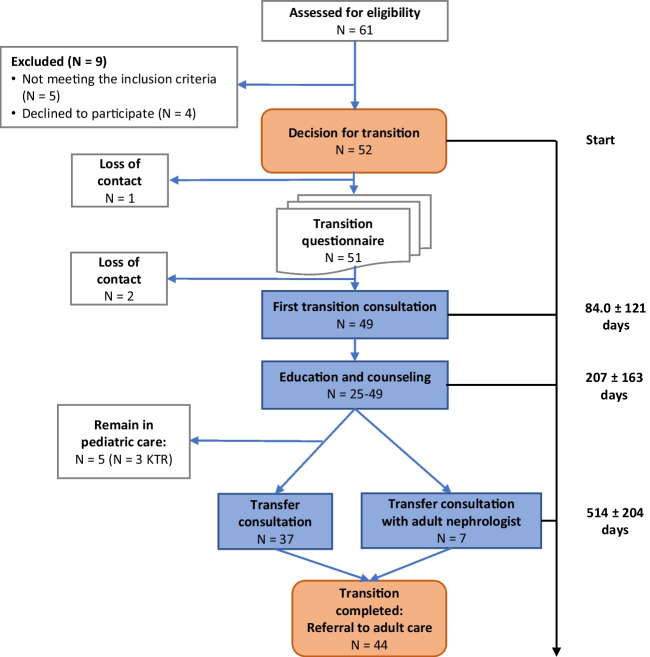
Flow chart of the transition pathway. For the description, see the text

Patient characteristics and primary kidney diseases are shown in Table 1. Others include one KTR with primary hyperoxaluria type 1 and two patients with pre-transplant CKD suffering from the infantile nephrotic cystinosis and from the Lowe syndrome. From a total of 61 patients, 52 patients (37 males, 15 females) with CKD were eligible according to the inclusion and exclusion criteria and underwent the structured transition pathway (Fig. 1). Of the 9 patients excluded, 5 patients did not meet the inclusion criteria and 4 patients, including 1 patient with CKD 4 and 1 patient with CKD 5, declined to participate. The reasons for this decline were concerns regarding data protection. Twenty-nine of the 52 adolescents (55.8%) were KTR, and 23 (44.2%) were patients with pre-transplant CKD. Ten patients were in CKD stage 1, 6 patients in CKD stage 2, 5 patients in CKD stage 3, and one patient each in CKD stages 4 and 5. The mean age at enrollment was 18.7 ± 1.8 years.

**Table 1:** Patient characteristics

	Entire cohort	Pre-transplant chronic kidney disease	Kidney transplant recipients	*P* value (CKD vs. KTR)
Number	52	23	29	> 0.99
Age at the start of transition (years)	18.7 ± 1.8	18.2 ± 1.4	19.1 ± 2.0	0.076
Age at transfer to adult care (years)	20.4 ± 2.0	19.4 ± 1.7	21.1 ± 1.9	0.0038
Male (%)	37 (71%)	17 (74%)	20 (69%)	> 0.7*
Primary kidney disease				
CAKUT	*n* = 23	*n* = 6	*n* = 17	0.003^+^
Tubulointerstitial nephritis and cystic kidney disease	*n* = 6	*n* = 1	*n* = 5	
Glomerulopathy	*n* = 15	*n* = 10	*n* = 5	
Tubulopathy	*n* = 5	*n* = 5	*n* = 0	
Other	*n* = 3	*n* = 1	*n* = 2	

Data are mean ± SD, if not indicated otherwise. *, using the chi-square test; ^+^, using Fisher’s exact test. ^+^Patients with glomerulopathy or tubulopathy were more frequent in the group of pre-transplant CKD. Patients with CAKUT or tubulointerstitial nephritis and cystic kidney disease were more frequent in the group of KTR

*CAKUT*, congenital anomalies of the kidney and urinary tract; *CKD*, chronic kidney disease; *KTR*, kidney transplant recipients

### Education and counseling

#### Knowledge assessment

**Fig. 2:**
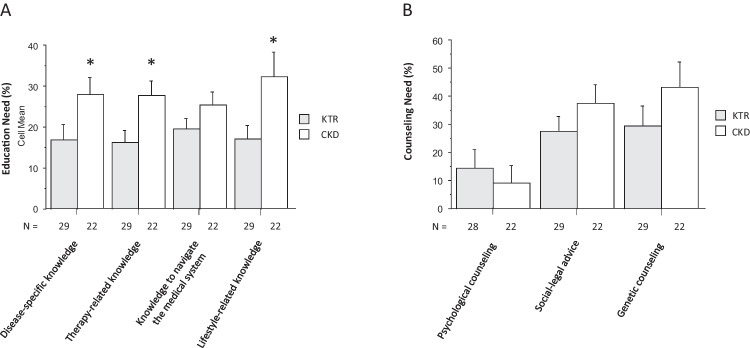
Educational (**A**) and counseling (**B**) needs of kidney transplant recipients (KTR) and pre-transplant patients with chronic kidney disease (CKD). *N* indicates the number of questions answered. Data are mean ± SD. Statistics by unpaired Student *t*-test, *P < 0.05 KTR vs. CKD

Self-assessed knowledge gaps (as indicated by the responses “partially agree; disagree”) were reported by 16–32% of patients; they were more frequent in patients with pre-transplant CKD than in KTR (Fig. 2A). These differences were significant for the items “disease-specific knowledge and need for education,” “therapy-related knowledge,” and “lifestyle-related knowledge,” while the difference between patients with pre-transplant CKD and KTR regarding the ability to navigate the medical system independently was less pronounced. Both groups had a similar self-assessed need for social-legal counseling (28–38%) and genetic counseling (29–43%) and a low self-assessed need for psychological counseling (9–14%) (Fig. 2B).

#### Educational sessions

**Fig. 3:**
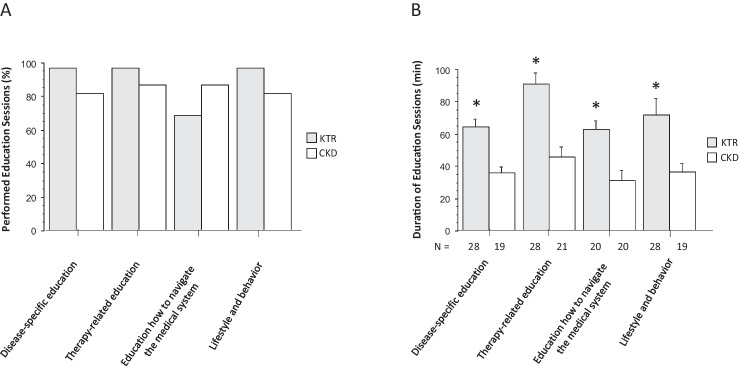
Number (**A**) and duration (**B**) of educational sessions for kidney transplant recipients (KTR) and pre-transplant patients with chronic kidney disease (CKD). *N* indicates the number of educational sessions performed. Data are mean ± SD. Statistics by unpaired Student *t*-test, *P < 0.05 KTR vs. CKD

A median of 3 (range 2–7) educational or counseling sessions was provided to patients with pre-transplant CKD, and a median of 5 (range 1–7) sessions was provided to KTR (Table 2). Counseling was tailored to the individual needs of each patient, as assessed by the patient questionnaire. The number of counselors in the multidisciplinary team (physicians, psychologists, social workers, dieticians, nurses) varied from 1 to 5 (median, 3 team members) (Table 2). Patients with pre-transplant CKD received education on disease-specific medication and on independence in 87% and on disease and lifestyle in 82% (Fig. 3A). From all KTR, 97% received education on the specifics of their disease, disease-specific medications, and lifestyle and behavioral aspects; 69% received education on how to navigate the medical system (independence) (Fig. 3A). The average duration of each specific education session ranged from 31 to 90 min (Fig. 3B). In all four categories, education sessions were significantly longer in KTR than in patients with CKD (Fig. 3B).

**Table 2:** Consumption of resources: number and duration of consultations, number of participating team members, and required time for preparing the epicrisis and for coordination

	Entire cohort (*n* = 52)	Pre-transplant chronic kidney disease (*n* = 23)	Kidney transplant recipients (*n* = 29)	*P* value (CKD vs. KTR)
Number of consultations				
All transition consultations	Median 5 (range, 1–7)	Median 3 (range, 2–7)	Median 5 (range, 1–7)	0.0005
Transfer consultations	1 (*n* = 44)	1 (*n* = 18)	1 (*n* = 26)	> 0.99
Transfer consultation with an adult nephrologist	1 (*n* = 7)	1 (*n* = 1)	1 (*n* = 6)	n.a
Duration of consultations (min)				
All transition consultations	96.3 ± 41.5 (range, 30–220)	82.6 ± 33.6 (range, 45–210)	103 ± 43.3 (range, 30–220)	0.0009
Transfer consultations	94.0 ± 47.8 (range, 30–210)	88.3 ± 45.7 (range, 45–210)	97.9 ± 49.8 (range, 30–200)	0.52
Transfer consultation with an adult nephrologist	80.0 ± 28.3 (range, 60–120)	60	83.3 ± 29.4 (range, 60–120)	n.a
Participating team members				
Total transition consultations	Median 3 (range, 1–5)	Median 3 (range, 2–4)	Median 3 (range, 1–5)	0.29
Transfer consultations	Median 3 (range, 1–5)	Median 3 (range, 2–4)	Median 3 (range, 1–5)	0.78
Transfer consultation with an adult nephrologist	Median 2 (range, 2–3)	2	Median 2 (range, 2–3)	n.a
Administration				
Time for compiling the epicrisis (min)	94.1 ± 31.5 (range, 40–180)	71.0 ± 19.7 (range, 40–120)	112 ± 27.0 (range, 60–180)	< 0.0001
Time for coordination (min)	200 ± 47.1 (range, 130–330)	165 ± 25.5 (range, 130–230)	227 ± 41.9 (range, 180–330)	< 0.0001

Data are mean ± SD (range) or median (range)

*CKD*, chronic kidney disease; *KTR*, kidney transplant recipients; *n.a.*, not applicable

#### Counseling sessions

**Fig. 4:**
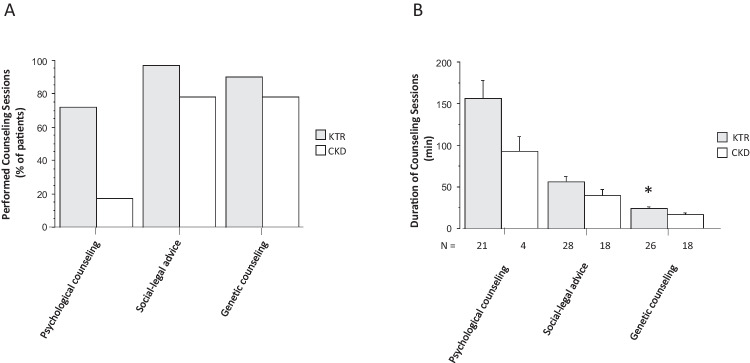
Number (**A**) and duration (**B**) of counseling sessions for kidney transplant recipients (KTR) and pre-transplant patients with chronic kidney disease (CKD). *N* indicates the number of counseling sessions provided. Data are mean ± SD. Statistics by unpaired Student *t*-test, *P < 0.05 KTR vs. CKD

Legal and genetic aspects were frequently discussed in patients with pre-transplant CKD and KTR, but psychological counseling was less frequent, especially in patients with pre-transplant CKD (17%) (Fig. 4A). However, when performed, it took the most time, especially in KTR (mean, 156 ± 28 min; range, 50–400 min) (Fig. 4B).

### Transfer to adult care

Of the 52 patients initially enrolled, 44 (84.6%) completed the entire transition pathway (Fig. 1). The mean age at transfer was 20.4 ± 2.0 years (range, 17.3–25.6 years) (Table 1). The mean time from the decision to start the transition process to the final transfer consultation was 514 ± 204 days (Fig. 1). The duration of the entire process was significantly longer for KTR (624 ± 150 [range, 307–819] days) than for patients with pre-transplant CKD (365 ± 172 [range, 0–693] days; *P* < 0.0001). Consequently, the mean age at transfer to adult care was higher in KTR (21.1 ± 1.9 years) than in patients with pre-transplant CKD (19.4 ± 1.7 years) (*P* = 0.076) (Table 1).

Transfer consultations were performed for 44 patients, but only for 7 patients jointly with the future adult nephrologist (Table 2). Most consultations were performed by the multidisciplinary team with a median of 3 team members. Consultations lasted 102 ± 43.3 min for KTR and 82.6 ± 33.6 min for patients with pre-transplant CKD (*P* = 0.0009) (Table 2). Mean transfer consultation durations with the future adult nephrologist were 83.3 ± 29.4 min for KTR and 60 min for one patient with pre-transplant CKD (Table 2). The median number of participating multidisciplinary team members was 2. At the end of the transition process, 44 patients were transferred to adult care and 5 patients (3 KTR) remained in pediatric care (Fig. 1), because no adult health care specialist could be identified. Reasons were severe neurological retardation (*n* = 2 Mainzer–Saldino syndrome, *n* = 1 Galloway–Mowat syndrome) or complex metabolic diseases (primary hyperoxaluria type 1 (*n* = 1) and infantile nephrotic cystinosis (*n* = 1).

### Administration

A central document in the transition process is the preparation of an epicrisis; the mean time required to prepare this epicrisis was 94.1 ± 31.5 min (Table 2). Compilation of the epicrisis for KTR (112 ± 27 min (range, 60–180)) was significantly more time-consuming than for patients with pre-transplant CKD (71.0 ± 19.7 min (range, 40–120)) (*P* < 0.0001) (Table 2). The case manager structured the transition process. Initially, the distribution and later the evaluation of the questionnaires were essential to assess the educational needs. The case manager was also responsible for scheduling outpatient visits, inviting the necessary members of the multidisciplinary team, and communicating with the patients and their families. This organizational work required an average of 200 ± 47.1 min (range, 130–330); it was more time-consuming for KTR (227 ± 41.9 min (range, 180–330) than for patients with pre-transplant CKD (165 ± 25.5 min (range, 130–230; *P* < 0.0001) (Table 2).

### Costs of counseling sessions and team members

The mean cumulative cost of all counseling and education sessions performed including the transfer counseling was 763 ± 473 Euro; it was significantly higher in KTR (966 ± 457 Euro) than in patients with pre-transplant CKD (470 ± 320 Euro; *P* < 0.0001) (Table 3). The cost per consultation was also significantly higher in KTR (192 ± 84.8 Euro) than in patients with pre-transplant CKD (130 ± 60.3 Euro; *P* = 0.0072). Regarding the costs of the multidisciplinary team members, the costs for the pediatric nephrologist were the highest, followed by the costs for the psychologist, the nurse, the social worker, the dietician, and the adult nephrologist. The costs of compiling the epicrisis (80.6 ± 19.4 Euro vs. 51.1 ± 14.2 Euro; *P* < 0.0001) and coordinating the transition process (118 ± 21.8 Euro vs. 85.7 ± 13.2 Euro; *P* < 0.0001) were significantly higher in KTR than in patients with pre-transplant CKD (Table 3).

**Table 3:** Cost of resources: cost of the participating team members during consultations, cost of preparing the epicrisis, and cost of coordination

	Entire cohort (*n* = 52)	Pre-transplant chronic kidney disease (*n* = 23)	Kidney transplant recipients (*n* = 29)	*P* value (CKD vs. KTR)
Cost of consultations (Euro)				
Total transition consultations	763 ± 472 (range, 118–2213) *n* = 49	470 ± 320 (range, 118–1335) *n* = 20	966 ± 457 (range, 170–2213) *n* = 29	< 0.0001
Per transition consultation	167 ± 81.2 (range, 59–443) *n* = 49	130 ± 60.3 (range, 59–312) *n* = 20	192 ± 84.8 (range, 81–443) *n* = 29	0.0072
Transfer consultations	175 ± 127 (range, 28.8–554) *n* = 44	159 ± 118 (range, 53.1–481) *n* = 18	185 ± 134 (range, 28.8–554) *n* = 26	0.50
Transfer consultation with an adult nephrologist	119 ± 38.8 (range, 86.4–173) *n* = 7	125 ± 39.4 (range, 86.4–173) *n* = 6	86.4 *n* = 1	n.a
Cost of participating team members during all consultations (Euro)				
Pediatric nephrologist	306 ± 155 (range, 72–641) *n* = 49	200 ± 120 (range, 72–540) *n* = 20	380 ± 134 (range, 72–641) *n* = 29	< 0.0001
Adult nephrologist	87.4 ± 46.0 (range, 43.2–166) *n* = 7	43.2 *n* = 1	94.8 ± 45.6 (range, 43.2–166) *n* = 6	n.a
Psychologist	198 ± 123 (range, 59–448) *n* = 25	150 ± 92.7 (range, 64.9–260) *n* = 4	207 ± 128 (range, 59–448) *n* = 21	0.41
Dietician	104 ± 92.6 (range, 33.6–269) *n* = 9	59.2 ± 22.7 (range, 33.6–76.8) *n* = 3	125 ± 109 (range, 43.2–269) *n* = 6	0.35
Nurse	189 ± 94.0 (range, 46.0–409) *n* = 49	126 ± 72.7 (range, 46–317) *n* = 20	233 ± 82.3 (range, 46–409) *n* = 29	< 0.0001
Social worker	141 ± 83.2 (range, 39–463) *n* = 47	113 ± 58.2 (range, 39–239) *n* = 18	159 ± 92.1 (range, 46.8–463) *n* = 29	0.06
Cost of participating team members during transfer consultations (Euro)				
Pediatric nephrologist	67.7 ± 34.4 (range, 21.6–151) *n* = 44	63.6 ± 32.9 (range, 32.4–151) *n* = 18	70.5 ± 34.4 (range, 21.6–144) *n* = 26	0.52
Psychologist	100 ± 16.2 (range, 70.8–124) *n* = 9	106 ± 25.0 (range, 88.5–124) *n* = 2	98.6 ± 15.1 (range, 70.8–118) *n* = 7	0.59
Dietician	86.4 ± 13.6 (range, 76.8–96) *n* = 2	76.8 *n* = 1	96.0 *n* = 1	n.a
Nurse	45.4 ± 21.9 (range, 13.8–96.6) *n* = 40	40.6 ± 21.0 (range, 20.7–96.6) *n* = 18	49.3 ± 22.3 (range, 13.8–92.0) *n* = 22	0.21
Social worker	52.1 ± 24.3 (range, 23.4–109) *n* = 33	53.4 ± 24.1 (range, 26–109) *n* = 13	51.4 ± 25.1 (range, 23.4–104) *n* = 20	0.82
Cost of participating team members during transfer consultations with an adult nephrologist (Euro)				
Pediatric nephrologist	57.6 ± 20.4 (range, 43.2–86.4) *n* = 7	43.2 *n* = 1	60.0 ± 21.2 (range, 43.2–86.4) *n* = 6	n.a
Adult nephrologist	57.6 ± 20.4 (range, 43.2–86.4) *n* = 7	43.2 *n* = 1	60.0 ± 21.2 (range, 43.2–86.4) *n* = 6	n.a
Nurse	27.6 *n* = 1	-	27.6 *n* = 1	-
Administration (Euro)				
Cost of compiling the epicrisis	67.8 ± 22.7 (range, 28.8–130) *n* = 46	51.1 ± 14.2 (range, 28.8–86.4) *n* = 20	80.6 ± 19.4 (range, 43.2– 130) *n* = 26	< 0.0001
Cost of coordination	104 ± 24.5 (range, 67.6–172) *n* = 45	85.7 ± 13.2 (range, 67.6–120) *n* = 19	118 ± 21.8 (range, 93.6–172) *n* = 26	< 0.0001

Data are mean ± SD (range)

## Discussion

This is the first study to precisely quantify the time required for the different components of the transition process in patients with pre-transplant CKD and KTR and to estimate the total and the proportionate personnel costs for the different members of the multidisciplinary team. The transition process took between 307 and 819 days, on average 70% longer in KTR than in patients with pre-transplant CKD. Overall, the transition was more resource-consuming for KTR than for patients with pre-transplant CKD: education sessions for all four categories studied—disease-specific education, therapy-related education, education on navigating the medical system, and education on lifestyle and behavior—took approximately twice as long as for patients with pre-transplant CKD. Transfer consultations also took about 20% longer in KTR than in patients with pre-terminal CKD. This result is not entirely unexpected, as a kidney transplant recipient usually has more complex medical problems than a patient with CKD before the need for chronic dialysis therapy. In addition, most CKD patients were in a low CKD stage, which is usually associated with fewer educational needs and less need for psychological counseling. On the other hand, self-assessed knowledge gaps were less frequent in KTR than in patients with pre-transplant CKD, probably because these patients have been chronically ill for a longer period of time and have therefore been dealing with the self-management of CKD for a longer period of time. Administrative management of the transition was also more time-consuming for KTR (3.8 h) than for patients with pre-transplant CKD (2.8 h). This structured transition pathway for adolescents with CKD resulted in 84.6% of patients successfully transitioning to adult care, while 9.6% remained in pediatric care; 5.8% were lost to follow-up.

At the beginning of the transition process counseling needs were assessed using a standardized questionnaire [16]. In our study, the self-assessed knowledge gaps were generally quite small, but more present in patients with pre-transplant CKD than in KTR. However, even if the self-reported knowledge gaps are small, effective education sessions should be provided for all patients, especially to adolescent KTR due to their more complex medical condition. According to the current consensus guidelines, repeated and regular educational sessions are recommended when necessary [8, 10, 20]. Thus, education is a critical part of the transition as it improves patient empowerment [21–24]. Thus, empowerment is strongly associated with self-management, and self-management improves health outcomes in chronic disease, not only by improving adherence to the treatment plan, but also by increasing the individual’s ability to overcome challenges and solve problems [24].

The large discrepancy between the results of the questionnaires and the education and counseling sessions provided suggests that patients tend to overestimate their level of health literacy. For example, few requests for psychological counseling were reported in the questionnaire. This finding contradicts the high frequency (72%) of psychological counseling sessions with a mean duration of 156 min among adolescent KTR. This discrepancy can be explained by the negation of psychological problems in adolescence as a difficult period of life with a tendency towards inappropriate help-seeking [25].

Of particular interest in our study was the calculation of costs for the entire transition process, excluding regular medical care such as physical examination, laboratory testing, and radiological examinations, which are also provided in these consultations. The mean cumulative cost of all counseling and education sessions performed, including the transfer counseling, was 966 ± 457 Euro for KTR and 469 ± 320 Euro for patients with pre-transplant CKD. Most CKD patients enrolled were in a low CKD stage which is usually associated with a smaller need for administrative work and lower costs. Two patients in an advanced stage of CKD (stages 4 or 5) declined to participate due to concerns regarding data protection. It is quite conceivable that the transition for patients in an advanced stage of CKD is similarly complex and resource-consuming as the transition of patients after kidney transplantation. However, since only a few patients with advanced CKD were included in our study population, we could not investigate this aspect. The mean costs of 500–1000 Euro per patient for the entire transition process are a relatively small amount compared to the costs of other medical interventions, e.g., 1 day of inpatient care at a university hospital (1500 Euro). In our study, the mean age at the start of the transition process was 18.7 ± 1.8 years, which may appear quite late. According to national and international consensus guidelines, the transition should be initiated at 12–14 years of age, and education and counseling as part of the transition process should begin at approximately 16 years of age [10, 26]. Considering that the transition process takes 2–4 years longer and that this would increase the costs by a factor of 2–4, the associated costs are still quite manageable. It is unreasonable that the costs of the transition are not yet covered by standard health insurances in many countries. It is well documented in the literature that the implementation of an integrated transition clinic, coupled with improving the health care experience of young adults through a young adult clinic, improves patient adherence to regular medication and engagement with health care providers, as measured by reduced rates of kidney transplant failure [27]. Ultimately, a successful transition process is cost-effective, because costly medical complications such as rejection or graft loss can be avoided. It is gratifying that the transition program of the project TRANSLATE-NAMSE succeeded in obtaining financial reimbursement from at least some German insurance companies in the year 2022. However, there is still a lack of nationwide funding for the transition of adolescents with chronic diseases to adult care in Germany.

The strength of this study is that, for the first time, it evaluated an established transition pathway for patients with CKD and calculated the time and personnel as well as financial resources required for education, counseling, and transfer sessions. One limitation is the lack of long-term follow-up data, because the funding of the TRANSLATE-NAMSE project was limited to 3 years. Furthermore, this study was a single-center study of the University Hospital Heidelberg; it is therefore probably not representative of all German centers or of a more global perspective. The calculated personnel costs are only valid for Germany and could be quite different for non-EU countries. Another limitation of this study is that 70% of our CKD patients were stages 1–2. Patients who have CKD stage 5 and nearing the need for dialysis or transplant require careful planning around transition and transfer of care, and this would require much more time and associated personnel cost than a patient with CKD stage 1. However, since only a few patients with advanced CKD could be included in our study population, we could not investigate this aspect.

In conclusion, this study shows that a structured transition pathway for patients with CKD is time- and resource-consuming due to the complexity of the disease. Given that costs are reasonable, sustainable funding should be mandatory.

### Supplementary Information

Below is the link to the electronic supplementary material.

Supplementary file2 (PDF 1318 KB)

Supplementary file1 (DOCX 149 KB)

Graphical abstract (PPTX 82 KB)

## Data Availability

Additional data are available upon request from the corresponding author if in line with the consents.
