# Supplementary material for: Resource use and costs of transitioning from pediatric to adult care for patients with chronic kidney disease
Source: Pediatr Nephrol. 2023 Jul 19;39(1):251–60. doi: 10.1007/s00467-023-06075-w (PMC10673743; doi:10.1007/s00467-023-06075-w)
Supplement: Supplementary file 1 — Supplementary file2 (PDF 1318 KB) [file 467_2023_6075_MOESM1_ESM.docx]

**Supplemental Material**

**Supplemental Table 1.** Standardized transition questionnaire

**Evaluation of Education need**

| **Disease-specific** | **Therapy-related** | **Medical system** | **Lifestyle** |
| --- | --- | --- | --- |
| I know the name of my condition and can explain it to others. | I am familiar with the different aspects of my treatment. | If necessary: I have an emergency card and carry it with me. | I am familiar with potential consequences of my condition on sexuality and contraception. |
| I understand which examinations and tests are being done and why. | I know the names of my medications. | I receive sufficient support from my partner, my parents, or other adult confidants. | I am familiar with the potential impact of tobacco, alcohol, and other drugs on my condition and treatment. |
| I know which symptoms and complications can occur in the course of my condition. | I am familiar with the effects and side effects of my treatment/medications. | I am close to an adult (friend or family), who is well informed about my condition and its treatment. | I know what to consider when preparing for travel. |
| I recognize signs of deterioration of my condition. | I take my medications independently, and/or carry out my treatment independently. | I feel adequately informed about support offers. | I am familiar with potential effects of my condition/treatment on my ability to obtain a driver’s license and drive a vehicle. |
| I am familiar with the effects of my condition/treatment in the event of pregnancy or childbirth. | I take my medications regularly, and/or carry out my treatment regularly. | I know how to get in contact with other people who have the same condition. | I know what my teacher/employer needs to know about my condition. |
| I would like the treatment team to provide more information about my illness. | I can adapt my medications/treatment as necessary to certain circumstances (e.g. stress, infection, leisure time). | During clinic appointments I speak to my doctor alone, or choose who accompanies me. | I understand the potential impact of my condition on my ability to work and take that into consideration when choosing a job. |
|  | I know which factors may influence the effect of my medications. | I can arrange my clinic appointments by myself. | I would like the treatment team to provide support for my questions about school, training, studies, and work. |
|  | I am familiar with necessary adjustments and information in the event of a surgical procedure. | I know how to contact my treatment team (e.g. doctors, diet consultants). |  |
|  |  | I can take order prescriptions/refills myself. |  |

**Evaluation of counseling need**

| **Psychological counseling** | **Social-legal counseling** | **Genetic counseling** |
| --- | --- | --- |
| I would like the treatment team to provide psychological counseling. | My condition or its treatment is a financial burden for me. | I understand the underlying genetic/inheritance rules of my condition. |
|  | I would like the treatment team to provide information on how to deal with authorities and legal changes after reaching the majority age of 18 years (e.g., health insurance, administrations, insurance). |  |

**For each surveyed point the patient should indicate which applies best**

| Agree | Partially agree | Disagree | Does not apply to my condition |
| --- | --- | --- | --- |

**Supplemental Table 2.** Average annual personal costs

| **Profession** | **Average annual personal costs (Euro)** | **Resulting costs (Euro) per minute** |
| --- | --- | --- |
| Pediatric or adult nephrologist | 111,300 | 0.72 |
| Psychologist | 75,400 | 0.59 |
| Social worker | 64,000 | 0.52 |
| Dietician | 61,150 | 0.48 |
| Nurse | 58,300 | 0.46 |

The basis for the calculation of the personnel costs was the TV-Ärzte (Unikliniken) Tarifgemeinschaft deutscher Länder (TVÄ-TdL) for the years 2017–2019 (https://www.oeffentlichen-dienst.de/entgelttabelle/tv-l.html). The medical professions were classified in the corresponding salary group as follows: pediatric or adult nephrologist (Ä2 level 4), psychologist (E13), social worker (E10), dietician (0.5 E9/0.5 E10), nurse (E9).

**Supplemental Figure 1.** Flow chart of the transition pathway

**
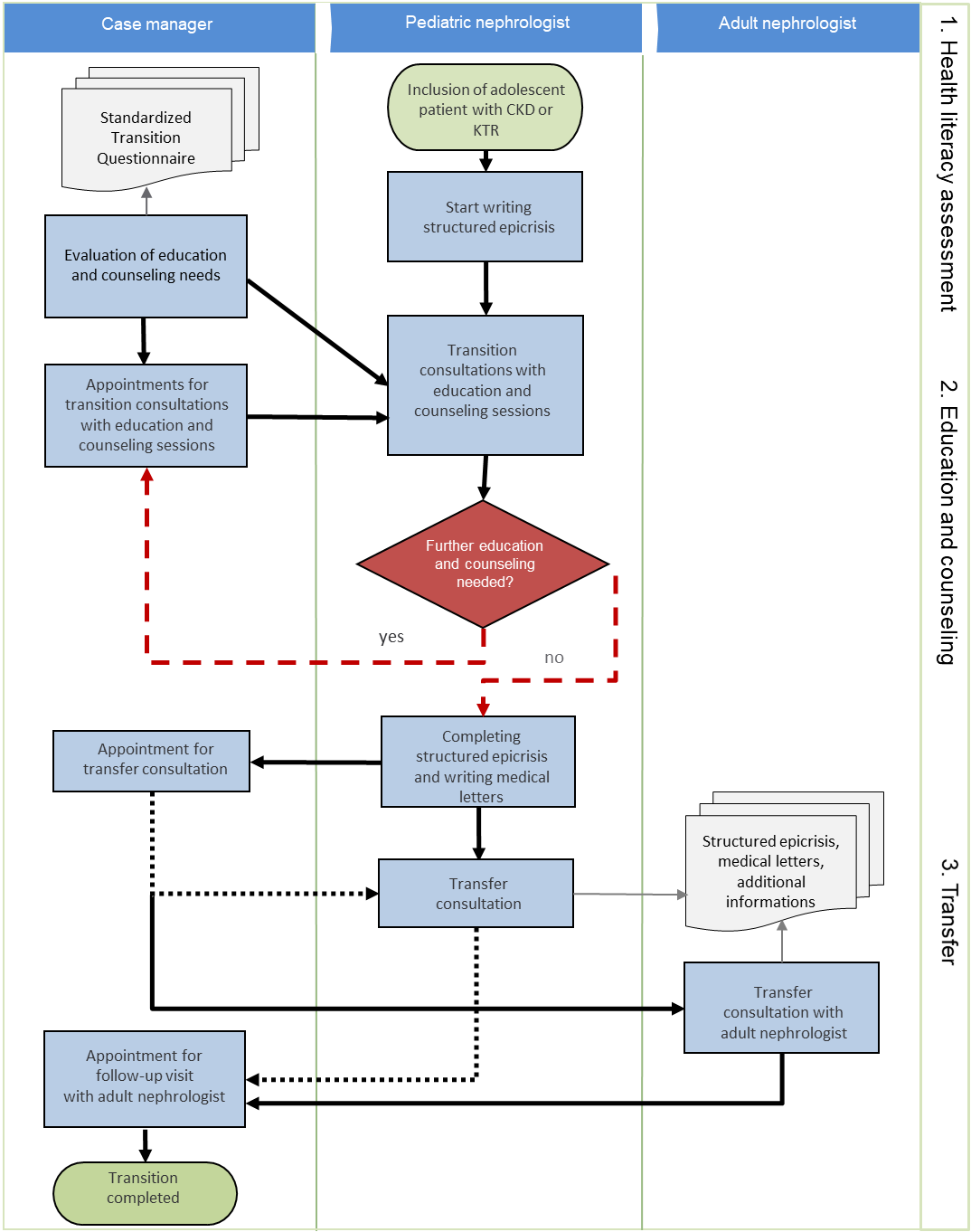
**
