# Supplementary material for: Resource use and costs of transitioning from pediatric to adult care for patients with chronic kidney disease
Source: Pediatr Nephrol. 2023 Jul 19;39(1):251–60. doi: 10.1007/s00467-023-06075-w (PMC10673743; doi:10.1007/s00467-023-06075-w)
Supplement: Supplementary file 3 — Graphical abstract (PPTX 82 KB) [file 467_2023_6075_MOESM3_ESM.pptx]

## Slide 1
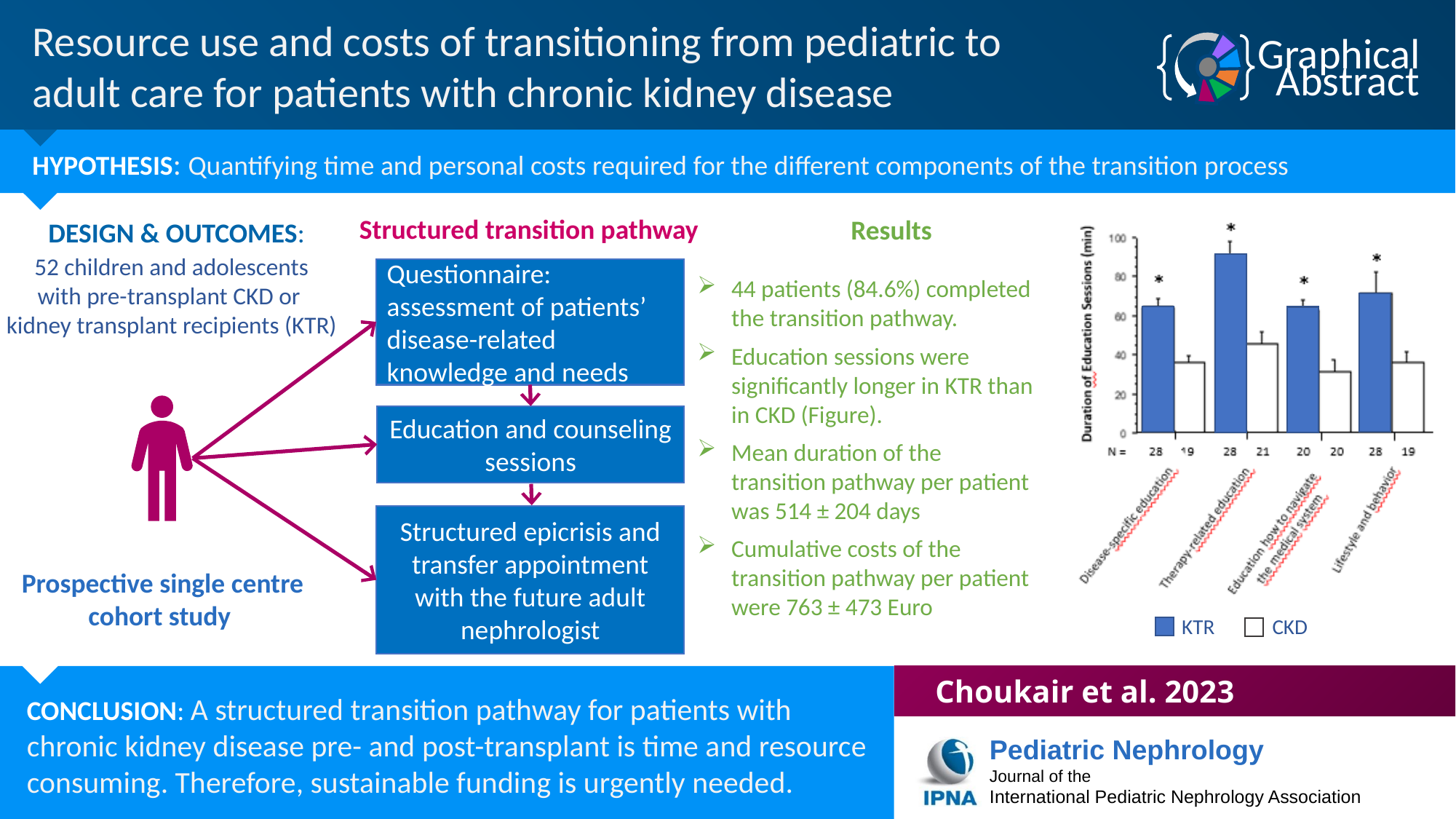

Resource use and costs of transitioning from pediatric to adult care for patients with chronic kidney disease
HYPOTHESIS: Quantifying time and personal costs required for the different components of the transition process
Structured transition pathway
Results
DESIGN & OUTCOMES:
52 children and adolescents
with pre-transplant CKD or
kidney transplant recipients (KTR)
Questionnaire: assessment of patients’ disease-related knowledge and needs
44 patients (84.6%) completed the transition pathway.
Education sessions were significantly longer in KTR than in CKD (Figure).
Mean duration of the transition pathway per patient was 514 ± 204 days
Cumulative costs of the transition pathway per patient were 763 ± 473 Euro
Education and counseling sessions
Structured epicrisis and transfer appointment with the future adult nephrologist
Prospective single centre
cohort study
KTR
CKD
 Choukair et al. 2023
CONCLUSION: A structured transition pathway for patients with chronic kidney disease pre- and post-transplant is time and resource consuming. Therefore, sustainable funding is urgently needed.
